# Health dynamics in camps and on campuses: stressors and coping strategies for wellbeing among labourers and students in Cameroon

**DOI:** 10.1080/17482631.2018.1435098

**Published:** 2018-02-13

**Authors:** Valerie Makoge, Harro Maat, Lenneke Vaandrager, Maria Koelen

**Affiliations:** a Health and Society (HSO) group, Wageningen University, Wageningen, The Netherlands; b Medical Research Centre, Institute of Medical Research and Medicinal Plant Studies (IMPM), Yaoundé, Cameroon; c Knowledge Technology and Innovation (KTI) group, Wageningen University, Wageningen, The Netherlands

**Keywords:** Poverty-related diseases, Cameroon, Cameroon Development Corporation, salutogenesis, University of Buea, University of Yaoundé

## Abstract

**Purpose:** For many people living in low-income countries, poverty implies an increased exposure to conditions that threaten health and wellbeing as well as reduced capacity to maintain health. Despite the challenging conditions caused by poverty, people may consider themselves healthy because they have learned to cope with their situation probably as a result of life experiences which expose people to both challenges and potential solutions. In this paper we present results from studying health and wellbeing challenges and mechanisms to cope with challenges among two different groups of people who are living under conditions of poverty: workers of the Cameroon Development Corporation (CDC) and students of the university of Buea and the university of Yaoundé.

**Methods:** We performed a cross-sectional study, interviewing 21 CDC workers and 21 students.

**Results** Our study reveals context-specific stressors emerging from poor work conditions and study pressure as well as non-context-specific stressors perceived by respondents as living conditions, poor healthcare and financial uncertainty. Respondents devised coping mechanisms to overcome exactly those stressors such as searching for additional money sources, preventive action towards hazardous living conditions and alternative medical support.

**Conclusion:** We conclude that supporting and promoting such avenues is essential for enhanced and continuous coping with stressors.

## Introduction

For many people living in low-income countries, poverty implies an increased exposure to conditions that threaten health and wellbeing (Singh & Singh, 2008). Poverty exposes people to more health threats, and reduces their capacity to maintain their health (Ningaye, Alexi, & Virginie, 2013). International agencies addressing poverty-related diseases (PRDs) focus mostly on the main infectious diseases (Singh & Singh, 2008). The World Health Organisation (WHO) classifies malaria, HIV and tuberculosis as major PRDs (WHO, 2012).

The connection between poverty and disease, obvious as it may seem, is not always straightforward. When, for example should a person be considered ill or healthy? According to the WHO, health is “a state of complete physical, mental and social well-being and not merely the absence of disease or infirmity” (WHO, 1948). This definition has not been modified since its conception over 60 years ago. Although seemingly inclusive, the WHO definition has been criticized for its projection of an ideal situation that few people can attain (Huber et al., 2011) and for ignoring the efforts that people make with respect to their challenges. For example, people have been shown to consider themselves very healthy in situations that others would characterize as great adversity (Bull, Mittelmark, & Kanyeka, 2013). Similarly, people with chronic diseases have shown tendencies to manage these diseases and live fulfilled lives (Cole & Reiss, 2013).

These points of critique formed the basis of our study on the relationship between poverty and health. In challenging situations caused by poverty, people may consider themselves healthy because they have learned to cope with their situation. In Cameroon, 40% of the population are considered as living below the poverty line (PNUD, 2006; World Bank, 2011). In this paper, we present results of studies on health and wellbeing challenges, and mechanisms to cope with these challenges, among two different groups of people who were living under conditions of poverty: employees (and their dependants) of the Cameroon Development Corporation (CDC) and students at the University of Buea and the University of Yaoundé. Both groups have income levels around the official poverty indicator of $1.90 a day. Using the salutogenic model of health (SMH), we investigated stressors and coping mechanisms of these two selected groups. Such an approach provides insights that are useful to target health promotion efforts geared towards people’s own capacities in places where official health services are defective and out of reach for the poor.

The SMH does not consider health and disease as a state or (permanent) condition but rather as a dynamic process. Antonovsky (1979) portrayed health as moving along a continuum, indicating, at one extreme, complete lack of health (dis-ease) and, at the other extreme, full health (ease). The salutogenic model therefore assumes that a wide variety of factors have an impact on the health–disease continuum. In our study, we focused on the different areas that have been reported to play a role in the health and wellbeing of people, such as living conditions, job, healthcare services, relationships and so on (Alnazly, 2016).

The SMH offers a platform for the identification and understanding of what people experience as stressors and resources. A key ability for a person is his/her capacity to comprehend and have an overview of a situation and foresee a way out. This follows from the person’s life experiences, i.e., a balance in life with respect to stressors and resources as well as having a life that is predictable and finally actively taking part in the shaping of his/her life (Bull et al., 2013). Such experiences are essential for putting in place what Antonovsky called Sense of Coherence (SOC). The SOC consists of three “senses”: a sense of what to expect in a given situation (comprehensibility), a sense that, in his or her situation, there are resources that can be employed (manageability) and a sense it is rewarding to act upon a situation (meaningfulness). The means available to people, termed General Resistance Resources (GRRs) and Specialized Resistance Resources (SRRs), can be material, biological, personal or social in nature. Having a strong SOC is linked to being more able to identify and use GRRs and SRRs (Eriksson & Lindström, 2011). The SMH thus considers a person’s own capacities in relation to health as a key resource and a crucial element in making other resources relevant in dealing with stressors. The presence of a stressor creates a tension that will cause people to shift in their position on the continuum either towards the health pole, when they overcome the challenges, or towards the dis-ease pole, when they fail to do so.

The aim of our study is to investigate the different types of stressors experienced by camp dwellers and university students and to explore their ability to draw on identified resources to circumvent life’s challenges. This information is essential for a better understanding of what health means and how people attain health in conditions of poverty (Bull et al., 2013). We sought to answer three questions: (1) What types of stressors are experienced by camp dwellers and university students? (2) What coping mechanisms do respondents use to deal with stressors affecting their health and wellbeing? (3) What recommendations can be given to increase resources for coping and reduce stressors experienced in the lives of our respondents?

The SMH has hardly been used in studies in developing countries. By using this model among two different groups of people in Cameroon, we provide a promising lens through which PRDs can be understood and managed.

## Methods

### Characteristics of the study settings and population

The study presented here was conducted in camps, the housing offered to workers of CDC, an agro-industrial company with plantations in the southwest region of Cameroon, and on campuses, the housing of students at the state universities of Buea and Yaoundé. These settings were selected because they are both home to people who originally came from different parts of Cameroon and moved to the settings for work or studies, respectively, and because respondents in both settings have a low income. The groups differ on various socio-economic characteristics, e.g., age, education and marital status.

CDC employs about 16,000 people, the majority of them being low-paid, low-educational-level wage labourers who live in camps with their families (CDC, 2009). Three camps were included in our study: Limbe camp, Camp 7 and Sonne camp. Camp 7 and Sonne camp have about 50 households each, whereas Limbe camp contains several hundred households. As a worker, a person together with his or her family is entitled to free healthcare services offered at CDC clinics or hospital, including consultation, laboratory analysis and free medication, if available.

The University of Buea (UB) has a population of over 12,000 students (UB, 2014), and University of Yaoundé (UNIYAO) has over 33,000 students (2007 estimate) (INS, 2010). For students of both universities few rooms are available on-campus, reason why most students live in neighbourhoods in the vicinity of the campuses. Students have to pay for healthcare themselves, off-campus and in cash.

### Study design and respondents’ characteristics

This study was cross-sectional in design and part of a larger study, with data collected over 2013 and 2014. We carried out semi-structured interviews with 21 camp dwellers and 21 students. Fourteen of the camp dwellers interviewed were male and seven were female. Fourteen of these had basic primary education, three had no formal education, three had secondary education and one had high school education. Twelve camp dwellers were married, six were single and three either separated or widowed. In the campus setting, 16 of the students were female and five were male, and all of them were single.

Camp dwellers who took part in this study were employed by CDC as rubber tappers, camp and compound cleaners, security guards or weeders. One camp dweller was a dependant of a CDC worker. Students interviewed were taking university courses such as educational psychology, journalism, diplomacy, English, life sciences and so forth.

### Procedure

Our results are based on observations in the camps and on campuses as well as on interviews with respondents. Camp dwellers were recruited in their houses in the camps, and students were recruited in their rooms in the student buildings. Camp dwellers were purposefully selected to reach different job categories while students were purposefully selected to reach different study programmes. Respondents were introduced to the researcher and the study protocol, and consent was sought for participation. Respondents’ anonymity was assured during this process. All respondents approached agreed to be interviewed. After 21 interviews in each setting, it appeared that no new information was forthcoming, therefore data collection stopped (Strauss & Corbin, 1998).

### Ethical considerations

This study was approved by the Wageningen University review board and CDC’s human resources and health departments. The aim and the procedure of this study were explained to all respondents who met the inclusion criteria. Respondents were informed of their right to leave at any stage without explanation. Respondents were assured of anonymity, and each respondent signed an informed consent form before participating in the study.

### Rationale for the salutogenic model of health

Our study employed the use of the SMH. The SMH studies health and determinants of health in the human frame of reference. This model has as primary goal to answer the question of what creates health by highlighting on how health is generated and the things and qualities that make people move towards improved or better health. In this study therefore, we sought to identify factors that hamper the health of respondents also known as stressors experienced and investigated ways employed to reduce the effects of these stressors on health.

### Interview guide

An interview guide was designed after preliminary visits to the camps and the campuses, conversations with health overseers in the camps and student leaders, and observations of activities in the settings. The interview guide was designed on the basis of the observations and the SMH (Antonovsky, 1979, 1987) and sought to identify stressors and coping mechanisms that made use of identified resources of camp dwellers and students. The first part of the guide contains background or entry questions ascertaining the respondents’ demographic characteristics. Then respondents were asked to rate their health as poor, fair, good or very good. What followed were questions to identify factors that challenge their daily lives (stressors), such as jobs for the workers and studies for the students and health for both groups. Probing enabled respondents to reflect on the resources on which they drew to manage these challenges. Most of the interviews were conducted in English. Pidgin-English was used in interviews when preferred in the camps. Interviews in the camps were conducted by the first author, assisted by a CDC head office junior worker. Interviews on the campuses were conducted by the first author helped by a trained student assistant.

### Data analysis

The interviews were audio-taped, and detailed field notes were also taken. Interviews in English were transcribed verbatim style by the first author. Pidgin-English interviews were translated into English as they were being transcribed. Data were analysed using the ATLAS.ti 7.5 software (Scientific Software Development). Thematic analysis was carried out following Braun and Clarke’s (Braun & Clarke, 2006) protocol. This protocol offers flexibility in identifying themes in various ways. The analysis process started with reading the transcripts several times for familiarisation with the data and then generating codes. The transcripts were imported into ATLAS.ti and coded. The data were coded top-down, guided by the SMH (Antonovsky, 1996). We identified themes encompassing stressors and coping mechanisms using identified resources (see Table I). Codes relating to workers’ jobs were for example: Job_risks and Job_workcon, and codes relating to academics were for example: Acad_lecturerstress and Acad_photocopyingstress.

**Table I.** Summary of themes and associated factors.

Categories following from the salutogenic model of health	Operationalisation of categories
1. Stressors experienced	Lack of space and poor living conditions Poor working conditions, low payment and intimidation Overload of social responsibilities and living up to family expectation Poor healthcare, healthcare inequality and lack of medication Uncertain remittances and financial problems
2. Drawing on resources for coping (coping mechanisms)	Perceived health Alternative ways to make additional money Maintaining social relationships Taking part in religious activities and faith Preventive action and improvisation

We also identified mechanisms through which respondents drew on resources to manage stressors, i.e., resistance resources (RR). An example is the theme “alternative ways to make additional money”. Examples of codes that fell under this category were: RR_farm, RR_business.

## Results

In this section, we start with a brief description of the context, followed by a description of the range of stressors experienced by the two different groups of respondents. Finally, we elaborate on mechanisms for coping with stressors.

The stressors experienced by respondents were classified under five main themes that emerged from the data analysis: lack of space and poor-living conditions; poor-working (study) conditions, low income and intimidation; overload of social responsibilities and living up to family expectations; poor healthcare, healthcare inequality and lack of medication; and uncertain remittances and financial problems. Table II provides an overview of these factors and how they are experienced. First, the results for camp respondents are presented and then those for campus respondents, where applicable.

**Table II.** Stressors experienced by camp dwellers and students and immediate consequences.

Stressor		The way it is experienced	Immediate consequences of the stressor
**Lack of space and poor living conditions**			
Accommodation	Camps	Insufficient space for family Insecurity and theft Poor hygiene conditions	Parents share bedrooms with grown children or convert kitchen to bedroom and cook outside Fear for one’s safety Increased risk of infection and diseases
	Campuses	Noise from neighbours	Difficulty in maintaining focus when studying
	Camps and campuses	Unhygienic conditions due to toilet sharing Neighbourhoods are conducive to the proliferation of mosquitoes	Rapid and easy spread of diseases such as vaginitis (commonly called sugar sugar) Frequent episodes of malaria
Water and electricity cuts	Camps	Frequent cuts in Limbe camp, rationing of light from 6pm to 10 pm in Sonne camp when available, no electricity installed in Camp 7 No permanent water supply	Cannot use fans or be entertained with radio or TV Forced to drink water from streams that are not very clean
	Campuses	Frequent electricity cuts Frequent water cuts and water of poor quality	Difficulty in maintaining focus and studying in darkness Forced to drink water from wells or buy water
**Poor working conditions, low payment and intimidation**			
Job-related	Camps	Abuse by job supervisors Wrong registration of hours Late retirement Job risks of accidents, injury, pain and death, lack of work equipment such as protective clothing, gloves, masks, cutlasses (agricultural tool) Poor working conditions such as low salaries, inhaling dangerous chemicals, no days off	Reduced work engagement Less salary at the end of the month Reduced ability to carry out other activities after retirement Fear for one’s safety and wellbeing Work is described as “hard”, “strenuous”, “difficult”, “not easy”; reduced work engagement
Study-related	Campuses	Sexual harassment of female students by male lecturers Unavailability of lecturers/supervisors Too many assignments Examination stress such as cross-setting exams and omission of marks on publication of results Poor study conditions such as small lecture halls, lecture halls not adapted for physically challenged students, language barrier, heavy financial demands	Mental trauma for the student Delay in completing programmesCampus life is described as “hell”, “a jungle”, “place to be when you are not there”, “a place whereby hypocrisy is encouraged”, a place in which there is favouritism, a difficult place to emerge or excel
Job and study-related	Both camps and campuses	Power relation stressors Lack of clean toilets in the camps and on the campuses	Reduced work or academic engagement, respectively Rapid and easy spread of diseases and infections
**Overload of social responsibilities and living up to family expectation**			
Family support	Camps	Marital conflicts Food insecurity Financial insecurity Difficulty experienced in taking care of children and unruly teenagers	Hampers health and wellbeing Unable to provide food for the family Unable to provide needs for the family
**Poor healthcare, health inequality and lack of medication**			
Healthcare services	Camps	Ambulance delay Health inequality Shortage of medication at CDC clinics Perceived poor quality treatment received at CDC clinics Money to seek treatment in non-CDC clinics or to buy medication	Death of workers Workers get worse treatment than management staff at clinics Need for frequent trips to clinic; need to buy medication outside CDC and also poor drug compliance as drugs are taken in half doses Leads to poor drug compliance and need to buy medication outside CDC Increase in self-medication practices Increase in self-medication practices
	Campuses	Inadequate support from parents and guardians Money needed for out-of-pocket payment for health services	Self-medication practices because students cannot afford to go to hospital and also their wellbeing hampered Increase in self-medication practices
**Uncertain remittances and financial problems**			
	Camps	For family For upkeep For healthcare	Hampers health and well being
	Campuses	For upkeep For better accommodation For healthcare For printing and photocopying	Hampers health and well-being

### Life in camps and on campuses

People in camps live a life that is ordered by the work regime of the plantation. They start their working day at 5 am with meetings where they hear general announcements and tasks are allocated for the day. They are then transported by trucks to the plantations where they carry out their expected tasks. Those who do not work in the plantations but in the camp compounds are also allocated tasks, which they perform in their camp or any other camp to which they have been assigned. After work, usually after midday, the workers are brought back by truck. Some go to their farms at this time, while others rest or engage in any other activity. In the event of ill-health, there is an office in each camp called the AID post, where mild illnesses are handled. A nurse who lives in the camp is in charge of this post. She has medications like pain killers and disinfectants for cuts that she can give to camp dwellers who need them. People who have illnesses that are beyond the competencies of the AID post are transferred to the clinic or hospital by a car (also called ambulance) that comes to pick up patients from the camps on certain days in the week.

Students live a life that is quite different from the people in the camps. They are younger and more educated, and all who took part in this study were single. Young and single camp dwellers have to take care of themselves, their children and their siblings. Among the student respondents, two mentioned being parents of at least one child. Most students still depend financially for their upkeep on their parents who are in different parts of the country. Students live in single rooms around the university premises. The students’ lives centre on their studies.

### Stressors

A stressor was operationalized as challenges faced by respondents for which they did not have an immediate response.

### Lack of space and poor living conditions

Life in the camps was characterized by congestion. Camp respondents indicated that factors that challenged their health and wellbeing were linked to accommodation space and poor living conditions. Respondents reported that many families lived a cramped lifestyle, often having to share bedroom space with their children. They also reported that the conditions in which they lived were deplorable. For example, heavy rainfall during the rainy season worsened living conditions.

*If you had walked up to my house you would feel sorry for me because water just comes into my home. When rain falls, no one can come into my house because it is usually flooded. The water gets up to waist level. You have to move in this water before going to the kitchen*. (Male camp respondent)

The students also reported that they lived in deplorable conditions. Landlords and neighbours were blamed for the state of students’ houses and poor hygiene conditions, respectively. They reported that their living environment was conducive to the proliferation of mosquitoes, with consequent frequent malaria attacks as well as the spread of other diseases.

*The rooms are cheap and dirty and the toilets are dirty and you can be sharing a toilet with 20 people. In the morning they form a line to use the bathroom and go in one after the other. …poverty makes people live in such conditions. When it rains, the water from the rain carries excrement because the people are opening their toilets to let excrement out. So when the water is passing in front of your house it is smelly and it can even enter your house*. (Male campus respondent)

Electricity and water cuts were common in both camp dwellers’ and students’ lives. In the camps, Limbe camp had a regular electricity supply. In Sonne camp, electricity was rationed. It came on between 6 pm and 10 pm, if it was available. Camp 7 had no electricity supply.

*We go sometimes for even three months without any light*. (Male camp respondent)

#### Poor work and study conditions, low income and intimidation

For camp dwellers, jobs played a central role in their daily lives. Even though having a job guarantees a salary at the end of the month, CDC workers were very discontent with their jobs and especially with the salary they received at the end of each month. The poor-working conditions that hampered their wellbeing were related to job risks, to lack of equipment for work and to treatment received from their bosses.

*The stress is with my boss. The way he acts with me makes me feel stressed out because the way I deal with people, I take everybody like a friend, like family, so when you are playing games with me, when I discover it, I feel bad*. (Male camp respondent)

*There are many risks. We can be contaminated with any disease. Also, there are many broken bottles around leading to the risk of cuts while cleaning*. (Female camp respondent)

As with camp dwellers’ jobs, the students’ studies had a central role to play in their daily lives. Students expressed strong feelings about what they thought was not going right at the university. The following quote sums up the general feeling of students:

*The major one is academic stress. For instance, if they give you an assignment for which you have to spend a lot for transportation, it stresses you out financially and the traffic you encounter also stresses. Thinking also about what is next after your degree* [future] *whether to look for a job or… it becomes more stressful*. (Female campus respondent)

Student life came with challenges both on- and off-campus and even extended to fears about the future. These challenges were likely to affect their wellbeing and productivity, as they observed. The major challenges reported were campus-related, examination-related, assignment-related and also lecturer-related. Most students had complaints about their lecturers. Lecturers were considered to be mostly unavailable, capable of sexual harassment, not motivating, inconsiderate and making things unnecessarily difficult.

*Life is a jungle on campus. People want to graduate but there are those who are trying to make sure they don’t* [lecturers]. (Male campus respondent)

Also, frustrations with lecturers were exacerbated by the large number of assignments the students had to do and which often required money. According to the students, lecturers frequently ordered them to repeat a task, and this made the financial burden heavier.

*There are some days that, when you go to school, each lecturer has an assignment for you to be submitted the next day in the morning and so you have to do like four assignments that same night … and so this gives me a lot of stress*. (Female campus respondent)

*…sometimes you spend the money and you are given a fail mark and asked to redo it and you have to spend some more money on redoing the assignment. When you call home for the money, or an uncle, and you have to call again for money for the same purpose, it looks like you were joking* [not serious]*…* (Male campus respondent)

Students also complained about the small lecture halls on campus and about the fact that the halls were not adapted for physically challenged students.

#### Overload of social responsibilities and living up to family expectations

An overload of social responsibilities derives from ways in which family, friends, neighbours and so forth can have a challenging impact on respondents’ lives.

Families can be strong sources of support, but dealing with them can also be challenging when there is an overload of social responsibilities. Camp dwellers experienced challenges taking care of their children because they had limited means and consequently not enough food for the family. Also, extended family obligations, marital conflicts and dealing with teenagers were reported to be challenging for parents.

*They* [children] *cause me stress because they are stubborn. You send them on errands and they don’t go and you have to repeat whatever over and over again…* (Female camp respondent)

*Family problems. Too many people needing me to intervene in their problems*. (Male camp respondent)

Unlike camp dwellers, students depended financially on their parents. Parents had expectations about their children completing their studies quickly and becoming independent. Because parents were far away, they were not always able to tend to the needs of their children regularly or as promptly as the students might need. Also, because students were facing a reality of which their parents were not aware, parents’ expectations could add to the pressure that students were under. Other social challenges adding to the emotional burden of students stemmed from intimate relationships. These challenges were likely to create an imbalance in the students’ lives that they had to manage.

*I have been told* [by parents] *that if I have a degree then I have to decide what to do. They say they will wash their hands of me and so this is like a blow to me because I have the desire to forge ahead with* [further my] *studies. As for now, I don’t know what I am going to do*. (Male campus respondent)

*He* [boyfriend] *is really a source of stress. At times when I need him, he is not just there*. (Female campus respondent)

#### Poor healthcare, healthcare inequality and lack of medication

Even though consultations and treatment are free, camp dwellers had very strong negative feelings about the services they were receiving. For example, it is routine for a car-“ambulance” to be sent to the different camps to pick up patients for the hospital on Mondays, Wednesdays and Fridays. However, this car could be late in coming, with potentially fatal consequences for severely ill workers.

*…We have lost people like that. We were waiting for the ambulance and it wastes a lot of time coming and the man dies. This has happened to about three people*. (Male camp respondent)

Also, workers reported experiencing health inequality in the services offered by CDC. In such cases, there was a perception that management staff received better quality treatment than the labourers.

*If a field assistant goes to the hospital, they would look for a carton* [box] *containing the real drugs and give them to them, which are necessary, but as workers, you go you will never receive this kind of treatment*. (Male camp respondent)

CDC has pharmacies with drugs for the patients who attend the clinics. However, it was reported that, when drugs run out of stock, they are not immediately replenished. In such cases, management staff would buy the medication themselves and be reimbursed, but the labourers would not be reimbursed if they bought medication. Furthermore, the drugs, when available, were reported to be of questionable quality for the field workers and not for the management staff. Medication for camp dwellers was reported to be administered sometimes without a laboratory diagnosis and with a poor attitude on the part of the medical staff.

*As for CDC, I don’t even want to think about them because, if you follow them, you will die. They don’t care for the workers. They say they give free medication but when you go* [to the CDC pharmacy], *the medication is unavailable. They write prescriptions that are continuously unavailable*. (Male camp respondent)

*The drugs that are given to you are not good first of all. They don’t give good drugs*. (Male camp respondent)

The consequences of taking drugs with severe side-effects would certainly affect the productivity of camp respondents. In addition, they reported that the one day sick leave to which they were entitled further hampered their productivity.

*One thing too, is that you are not given many days off as sick leave. You are given only one day and, the rest of days for which you have to drink the medicine, you are unproductive because of the side-effects*. (Male camp respondent)

Students reported that the main stressor in relation to healthcare was the money required to pay for hospital visits. As they did not have free medical services at their disposal, students were required to pay in advance and in cash for any medical services they needed. Because of this, there was a tendency to delay seeking medical help until they had tried self-medication or small pharmacy consultations and the illness still persisted and was therefore perceived as severe.

*…the real pharmacy* [medicine] *is expensive and, when I ask for a cheaper option, there usually isn’t and so I will now go back to the street vendors who will tell me they have something for a very cheap price and so that’s what I’d get*. (Female campus respondent)

#### Uncertain remittances and financial problems

The effect of limited finances was felt across many levels in the lives of camp dwellers and students. Camp respondents complained about having a small salary, which often was not enough for taking care of their immediate and extended families. In addition, they expressed uncertainty about the amount.

*Life is hard, things are very expensive and the salary is low*. (Male camp respondent)

Workers were paid per hour, and they were not always sure how many hours had been registered as work hours for them by their immediate supervisors/bosses. Such uncertainty created a feeling of despair among the workers, as can be seen in their comments with regard to their lives as CDC workers.

Students faced financial uncertainty as well, especially uncertainty about when parents would send money, and how much. Their financial needs spanned rents, accommodation facilities, upkeep, health and also academic demands (photocopying, printing and research).

*Financial problems again. Some people don’t have computers. All our assignments need to be typed, so it’s a problem of money to type here and come and give* [hand in] *at 7 o’clock. This is all boring and stressful*. (Male campus respondent)

Financial constraints were reported to reduce students’ quality of life. They were also perceived as a reason for increased sexual promiscuity, with the consequence of an increased risk of sexually transmitted infections.

*Students go out with a teacher because they want money and/or marks. They want somebody to take care of them by giving them money. Students go out with other rich students because they want money*. (Female campus respondent)

### Perceived health in the settings

Many stressors exist in the lives of camp dwellers and students. That notwithstanding, people reported being mostly in good health. People’s health was seen as a resource that enables them carry out their activities. Their responses indicated that good health was not only about how one feels physically, but also included mental or emotional considerations. Pains and headaches were considered to be a normal part of life. This implies that it was possible for respondents to say they were in good health even when they were physically in distress.

*Good health is feeling fine, being able to do anything, having the ability to do something. (*Male campus respondent)

The question which then follows from the above is what coping mechanisms would therefore ensure perceived good health and wellbeing in the face of the stressors that we have just reported? In the section below, we expatiate on respondents’ coping mechanisms.

### Drawing on resources for coping with stressors: coping mechanisms

Our study revealed several mechanisms through which respondents managed the challenges they faced. Some mechanisms encompassed a resource drawn upon to manage several stressors, whereas other mechanisms encompassed resources used to manage a specific stressor. Table III gives a detailed account of the resources available and how they are drawn upon by respondents. Below, we report on these mechanisms.

**Table III.** Coping mechanisms utilised by camp dwellers and students.

Strategies for overcoming stressors			
		**Resources**	**How they are drawn upon**
Alternative ways to make additional money	Camps	Farms	Planting, harvestings and selling produce in neighbouring markets
		Side-jobs	Sharpening skewers, part-time mechanic, business person
		Saving schemes	Being part of a njangi* group, credit union group
		Business ventures	Making skewers, selling fuel wood, selling groceries, selling food
		Borrowing money	‘10 for 13ʹ, borrowing 10 thousand to pay 13 thousand at the end of the month
	Campuses	Side-jobs	Teaching, baking, security guard
		Business	Selling shoes and clothing items
Maintaining social relationships	Camps	Family	Husband and wife support each other
		Neighbours	Sharing and helping one another out
		Church, village or traditional groups	Getting to know people, sharing, receiving help, learning new skills, saving money
		Youth groups in the camps	Receiving counsel from other group members and hanging out together
		Clubs	Meeting people, pursuing a shared vision, saving money
		Solidarity groups	Financial contributions to support a bereaved family
		Camp committee	A group of elected camp dwellers to oversee peace and security in the camps; camp committee resolves neighbourly conflicts
	Campuses	Family support	Parental support, support from extended family, siblings
		Neighbours	Sharing and mutual help
		Friends and classmates	Sharing handouts from lecturers with friends who had no money to photocopy; also sharing lecture notes with people who were absent
		Groups (church groups, *alma mater* groups, village groups)	Learning new skills such as music, public speaking, leadership skills, receiving help (financial or otherwise), getting to know people, saving money
		Other: boyfriends/girlfriends/sugar daddies	Moral and financial support
Taking part in religious activities and faith	Camps	Belonging to a church community or group, e.g., choir	Prayer, bible reading, saving schemes, moral and financial support, comfort
	Campuses	Being part of a church Being in the choir or a church-based youth group	Prayer, bible reading, learning, evangelism, social skills, music skills, receiving help, receiving counsel, being part of a group removes feelings of loneliness
Improvisational capacity and preventive action	Camps	Taking responsibility for work equipment	Workers bought gloves, masks, cutlasses (agricultural tools) etc. needed for their work
		Taking responsibility for timely repairs, e.g., public tap	Contributions to hire an external plumber for repairs of the common good
		Soap groups	Groups of camp dwellers who meet each week with a bar of soap that is handed to one person and the next week to another in a bid to cope with financial challenges
		Taking responsibility to increase space, e.g., extensions	To increase living space, extensions made of planks or zinc are attached to houses
		Loans	Collecting foodstuffs from shops and paying later
		Finding alternatives to medication with side-effects	Coping with side-effects of medication by drinking medication with soda Buying other medication from small pharmacies
		Finding alternative sources of water	Using alternative water sources such as plantation reserved water or streams Letting water settle before drinking
		Clean-up campaigns	Held every first Saturday of the month for the cleaning of camp premises
		Small pharmacies	Cheap alternative to CDC medication
		Herbs	Cheap and perceptibly effective alternative to CDC treatment
	Campuses	Finding a model	Knowledge of someone who has overcome difficulty before is inspirational in facing challenges; includes family relatives or others
		Assignments	Using free hour to catch up
		Taking responsibility to get an education: language barrier	Negotiating translations with invigilators Looking for opportunities to learn and speak French (friends)
		Taking responsibility to get an education: lecture halls	Students come early to ensure a space in the lecture hall Students wait outside lecture halls to collect notes from those who were present and photocopy them
		Finding a way to deal with parental expectation	Employing the services of guidance counsellors
		Quart-doctors [medical school students or graduate doctors without a place to practice yet]	Cheaper, more flexible in receiving payments, in close proximity
		Small pharmacies	Cheaper, in close proximity, available and affordable
		Herbs	Cheap and effective treatment against common diseases
		Clean-up efforts	Students focused on cleaning their micro-environments (room); some students in a student building once in a while gathered and cleaned the surroundings of the building
		Other ways of coping with campus-related challenges	Avoidance strategies, crying, sleeping, eating chocolates

#### Alternative ways to make additional money

Camp dwellers found several ways to augment their income. They looked for business opportunities that could bring in additional money, such as selling crops from their farms or basic everyday needs for camp dwellers. Also, they were involved in saving schemes commonly known as *njangi* and credit union schemes. Having side-jobs, such as making skewers, or borrowing money to pay back with interest (also known as ‘10 for 13ʹ) were mechanisms in place to augment income. Farming, for example, was a means not only to ensure food security, but also to make more money for other essential purchases.

*We are organized in that, when we face financial difficulties, the* [my] *wife will harvest some crops and sell in order to have money to buy fish, crayfish and other small things necessary for the house. So what is not sold we bring home and manage on it*. (Male camp respondent)

The students also reported looking for business opportunities. Baking and selling clothes and shoes to other students were ways to make additional money. Side-jobs such as part-time teaching were also reported, the income from which helped take care of students’ financial needs.

*I am also a teacher. I have students, I teach, I do private teaching. I have students whom I take… Teaching is a way to augment my income to take care of myself at the university*. (Female campus respondent)

#### Maintaining social relationships

Social relationships offered respondents practical, financial and/or emotional support. The responses showed clearly that both the camp dwellers and the students had a rich social network which was supportive in many ways. The social network ranged from immediate family members (parents and siblings) to extended family members (aunts, uncles and so forth) to neighbours, then neighbourhood groups and then church groups and traditional or village groups. It could be seen that these networks were important for the wellbeing of the respondents, who therefore had to maintain and care for these relationships so that they could continuously procure the expected benefits from them. A well-maintained relationship, for example between neighbours, ensured support in times of need.

*I have a good relationship with neighbours. I have been here for eight years and I have never quarrelled with my neighbours. This is because of the love that we have for one another, because when there are problems in their houses, I will intervene and when I have problems too, they will intervene*. (Male camp respondent)

Being part of groups such as church or neighbourhood groups also gave meaningful life experiences to camp dwellers, with multifaceted benefits.

*I joined because it* [neighbourhood group] *permits me to join with my neighbours once in a while and share some common ideas. We are organised in a way that, every week, we bring a bar of soap each and this helps us, because many people bring the bar of soap and one person takes it home and it goes around in turns like that. Also, if you are sick and in the hospital, contributions are made and given to you and, although they will not assist you totally, they are there for support and partial assistance. So there is a small financial benefit in it*. (Female camp respondent)

Students counted on support from their parents, older siblings and other family members like aunts and uncles, and they reported that these relationships had to be cared for so that they could continuously be supportive. For example, students were expected to perform well in their studies so that parents would continue paying for them.

*My parents are very supportive especially when it comes to education*. (Female campus respondent)

Relationships with friends, classmates, neighbours, youth groups, church groups, *alma mater* groups, village groups also required care and maintenance because of the practical (and sometimes disciplinary), emotional and financial benefits attached to them.

*I am part of YAS* [Young Amity Sisters] *because I like their aim, their goal. One of their aims is to help the less privileged. We visit the less privileged, we visit the sick in hospitals, we also protect our interests, we do njangi* [daily savings]. *Our savings are very compulsory … You have to make sure that, during every meeting day, you save so as to avoid paying a fine*. (Female campus respondent)

#### Taking part in religious activities and faith

A belief in God was highlighted as a major coping resource for respondents. Most camp dwellers and students who took part in this study were engaged in one religious-related activity or another. Their belief in God created an expectation of seeing positive changes in their circumstances or situations, and this was a motivating factor to engage in, and to keep, that relationship. Believing that God was in control gave respondents the feeling that the stressors they faced were manageable and worthy of engagement.

Camp dwellers were active members of church groups. Some were in the choir, others in women’s or men’s groups in the church, and they all confirmed having meaningful experiences (spiritual, moral, financial, learning or supportive) because they were part of such groups. Faith and hope were spiritual attributes that equipped respondents to engage their challenges.

…*when you hear the sermon, you will forget about your problems for a while and have hope in life that better things will come to you. Also, going to church, we learn through our friends. I belong to the CWF* [Christian Women’s Fellowship] *and there are things we have learned, such as overcoming shyness, and also being a peacemaker and resolving quarrels and conflicts. At first, I liked to get into quarrels, but now I am the trouble shooter*. (Female camp respondent)

For the students also, being active in a church group was seen as very helpful in solving a myriad of challenges including loneliness and financial, spiritual and moral issues.

*If you belong to a church and you have problems, they could pray for you in the church. If you are sick, or have financial problems, they would contribute and give you money. They could pray for you or could contribute. For example, somebody’s house burned down and they contributed to help him buy new furniture*. (Female campus respondent)

#### Improvisational capacity and preventive action

In this section, we report on specific mechanisms employed by respondents to address specific stressors. Such specific stressors were related to their work conditions (work equipment, repairs), study conditions (assignments, language barrier, lecture halls), living environment (lack of space, water, poor sanitation) and health (medication with side-effects in CDC, financial constraints for hospital-based care for the students) (see Table III for details).

CDC workers who understood the *modus operandi* of the company showed improvisational capacity and pro-activeness as a sign of being in control of their circumstances. For example, for tasks that required work equipment, CDC often failed to provide protective materials such as gloves, cutlasses (a type of agricultural cutting tool) or protective masks. Respondents reported buying these themselves or improvising in order to improve their working conditions.

*If it is not in stock, we buy to help ourselves*. (Female camp respondent)

*…I used my child’s towel to cover my nose, and put on my flip-flops to work and I had to work like that because, if I don’t work, I will not be paid even for two hours of work*. (Male camp respondent)

Improvisational capacity was also seen when camp dwellers went to the clinics and medication was unavailable. They reported active responses in which they used other possible options such as small pharmacies and herbal treatment.

*…when I see the street vendors passing by, I buy medication and take it*. (Male camp respondent)

For students, an important goal was to succeed academically despite any challenges. For example, Anglophone students who faced language barriers took preventive action to avoid failing by negotiating with invigilators for translations of the examination questions, which were set in French. They also improvised on ways to improve their mastery of the French language without taking formal classes.

*…reading books and watching French movies and documentaries, and also interacting since I hang out a lot with friends and I get to meet Francophones… and so I am forced to speak French. So with time, I improve my French*. (Female campus respondent)

Not all students were actively trying to cope with stressors. Actually, some students reported passive responses or coping styles towards study-related stressors. These could be described as emotional or avoidance responses to challenges. Abandoning the task, crying, eating chocolates and sleeping or eating were ways reported by several respondents for coping with the too many assignments they had to do.

Students’ improvisation for lack of access to professional doctors was seen in the employment of the services of quart-doctors (medical school students or recently graduated medical school students who are not yet legally practicing medicine). Also, as a substitute for expensive medication, students resorted to buying generic drugs from small pharmacies.

*…students have no money, they can’t borrow medicine from here* [hospitals]. *In that case, the students are forced to go to quart-doctors*. (Male campus respondent)

### Discussion and conclusion

Salutogenesis offered a platform on which we could highlight people’s efforts to cope with factors that challenge their health and wellbeing and to have meaningful life experiences. This aspect has been strongly ignored in the current fight against PRDs. Our study attempted to fill this gap by expatiating on how people manage the challenges in their lives. The purpose for outlining how people manage is not to show that respondents are well off. They still face many challenges that affect their health and wellbeing negatively and are aware of the poor-living conditions, and their consequences, with which they have to deal. By including in our study the mechanisms through which people deal with stressors, we offer a scope for rethinking health policies and providing health services to people living in poverty, as well as for rethinking social policies and areas to strengthen people’s capacities.

Four major insights emerge from our study. First, stressors are experienced by all respondents, some specific to each group and others common to both groups. Second, there is a thin line between stressors experienced by people and the resources on which they draw to overcome stressors, as sometimes what people draw on as resources could become what they experience as stressors and vice versa, e.g., work and studies for camp dwellers and students, respectively. Third, even in the presence of stressors, respondents report good health and meaningful life experiences. Fourth, respondents adopt mechanisms to cope with stressors to ensure that they live the lives they want, e.g., alternative ways to make additional money.

### Stressors experienced

Health and wellbeing are not limited to the healthcare system (Kumar & Preetha, 2012). People’s living environments, their daily activities and their relationships are potentially creating stressors that affect people’s health and wellbeing. Most stressors can have a negative or positive impact, depending on people’s capacity to manage them (Antonovsky, 1979).

Our study showed that many stressors experienced by respondents were linked to their immediate living environment. They complained of the poor hygiene conditions in which they lived, which were conducive to an increased disease risk. This indicates that interventions to fight against poverty and related diseases should have a special focus on hygiene conditions and ways to improve them. Moreover, having limited finances was experienced as a stressor in both settings. For example, the salary received by workers was reportedly insufficient to enable workers meet their family obligations. Students also complained of the heavy financial demands of their studies (e.g., printing and photocopying). People who cope with financial challenges are those who actively look for ways to deal with the consequences of these challenges. Our study revealed mechanisms through which people coped with challenges. In the case in point here, respondents were able to find alternative ways to augment their income as a coping mechanism, e.g., side-jobs and farming practices. This shows that respondents were actively participating in matters that concerned them (Eriksson & Lindström, 2011).

### Interchangeability between stressors and resources for coping

Another finding in this study is that there was a thin line between stressors experienced and resources on which people drew to cope. It was interesting to see that a factor could be a resource for coping at one time and at another time a stressor. This was seen relative to work (studies), to social relationships and to healthcare.

Work and studies were important aspects that gave meaning to the lives of camp dwellers and students, respectively (Awang, Amir, & Osman, 2013). However, the terms used to describe work or studies indicated that these were also a major source of stress (see Table II). Work was associated with low salary, inflicted pain and injury, and so forth, leading to a vicious relationship of poor job conditions, poverty and poor health (Stankevitz et al., 2016). Such tendencies have been confirmed by other studies (Awang et al., 2013; Munisamy, 2013). It is therefore in the interest of employers, such as CDC, to improve the working conditions of their employees and thus have a more productive labour force (Munisamy, 2013). Camp workers showed improvisational skills to make their working conditions better, e.g., using towels as protective masks, indicating an active understanding and management of a challenge.

Study-related stressors, e.g., assignments and financial burdens, were commonplace and the most highlighted by students. This has been confirmed by some studies (Verger et al., 2009) although others have pointed out that relationships (between students) are the main source of stress for students (Hurst, Baranik, & Daniel, 2013). In our study, the perceived high level of intimidation of students by lecturers was a prominent study-related stressor.

The two examples given above (work and studies) show that respondents participated actively in issues that concerned them. It also shows limitations in the services offered by organisations such as the CDC or universities. The pertinence of daily activities, work on plantations for the CDC workers and study for the students, as main sources of stressors indicates that employers, schools and universities have important roles to play in providing health and wellbeing. A healthy work or study climate clearly encompasses more than basic facilities or (free) healthcare services; it includes rethinking work (study) packages and task division. It is important for organisations to seek ways to restructure work or study in a way that benefits people’s health and wellbeing.

To delve further into the interchangeability between stressors and resources for coping, we underline social relationships such as family and neighbours, which are important sources of support. These relationships can also engender negative feelings of frustrations when the relationships are not properly maintained, e.g., in the case of an overload of social responsibilities for camp dwellers or failure to meet parents’ expectations for the students. In our study, students reported experiencing stress because their parents expected them to complete their studies on time and become independent. The study by Hurst, (Hurst et al., 2013) indicates that expectations of the students themselves, striving for perfection, created stress. Again, sources for stress also play out as potential coping mechanisms (Eriksson & Lindström, 2011).Our results show the influence of a rich social network in this regard and efforts made to maintain the network.

Last, in the camps, even though healthcare services were free for the CDC workers and their families, the hassles attached to them, such as poor medication with severe side-effects, poor attitude on the part of medical staff, constant unavailability of medication and so forth, gave people negative experiences when they used them but also elicited specific mechanisms to bypass the challenges imposed. Specific practices of self-medication and drug purchasing outside CDC were employed by camp dwellers to cope with the challenges.

The above examples emanating from our study show that stressors are everywhere and need to be managed. People’s capacity to manage stressors is seen in the ability to create balance in chaos. By creating alternative ways to make additional money, or by actively making an effort to maintain relationships, people are creating balance in their lives, i.e., they are making timely adjustments in their lives to reduce the unwanted aspects of a stressor by identifying and using available resources in order to live more fulfilling lives (Bull et al., 2013).

### Good health and well-being in the midst of stressors

Even in the presence of stressors, most respondents reported good health and meaningful life experiences. This was in part because of the social groups to which they belonged, which are instrumental in aiding respondents to cope with stressors. Studies have shown that stable social support is linked to a stable community and also to promoting a strong SOC (Sagy & Antonovsky, 1986; Sagy & Braun-Lewensohn, 2009). This richness of our respondents’ social networks (family, youth groups, church groups and so forth) was visible in this study and effective in enhancing coping. Religion has been reported to be a resource used to overcome great adversity (Manuti, Scardigno, & Mininni, 2016), and this was confirmed in our study. Even though a belief in God has been previous reported to be a way of avoidance coping in stressful situations (Laurin, Kay, & Moscovitch, 2008; Lazarus & Folkman, 1984), in our study respondents’ belief in God added meaning to their lives and equipped them to take active steps towards coping.

The capacity to deal with stressors is also facilitated when resistance resources such as GRRs are present. We were able to identify that resistance resources employed by respondents were either generalized resistance resources (GRRs), which are resources that address a multitude of stressors, or specialized resistance resources (SRRs), which are summoned to address a particular stressor. GRRs and SRRs enable people to perceive their lives as structured and organized (Skärsäter, Dencker, Bergbom, Häggström, & Fridlund, 2003). GRRs have been reported to ascertain the extent to which SRRs are available to people (Mittelmark et al., 2017). This is confirmed in our study when we see the way a coping mechanism such as finding alternative ways to make additional money can play out. Here, GRRs such as farming practices or having side-jobs lead to an increase in income and ensure or facilitate the use of SRRs, for example using small pharmacies, street vendors or quart-doctors (for the students) as a way to respond to diseases. SOC, GRRs and SRRs in combination therefore play a vital role in our respondents coping effectively (Volanen, Lahelma, Silventoinen, & Suominen, 2004) and explain the perception of good health in our study, even in the midst of stressors.

### Conclusions

The aim of our study was to investigate the different types of stressors experienced by camp-dwellers and university students and to explore their ability to draw on identified resources to circumvent life’s challenges. We employed the SMH whereby we focused on how respondents advance towards health despite the challenges they face. Our results showed that stressors were experienced by all respondents and whereas some stressors were context specific, others were common to both groups of respondents such as financial constraints. The presence of stressors did not prevent respondents from striving to have meaningful life experiences. Most prominently, our study revealed that respondent’s social infrastructure and religious beliefs and activities created a supportive environment around them which enabled them to cope. Our study therefore shows clearly that people are active participants in the shaping of their lives and offers a prominent lens through which poverty can be addressed.

### Study limitations

Our study was qualitative in nature. This excludes the potential of generalising our findings, by means of statistical calculations, to wider populations. However, the qualitative method and the theory underlying the Salutogenic Model of Health (SMH) allow us to indicate how the experiences of our respondents relate to the theory, articulate the mechanisms they employ to overcome stressors and therewith create a comparative case to studies using the same model.

### Future research directions

It would be interesting to carry out a similar study in different groups in the society and determine similar or different coping mechanisms with stressors.

### Recommendations

From the insights obtained in our study, we make the following recommendations:

- Improvements in the healthcare system and living conditions should pay more attention to adequate and reliable functioning of facilities. Improving the use and employment of limited means can be equally effective as bringing in new or additional equipment.
- Promotion and facilitation of social encounters should receive more attention in health policies, as our study has indicated enormous benefits from such encounters/activities.
- Facilitation by CDC for camp dwellers to have extra plots for farming should be considered so as to promote the planting of fruits and vegetables for consumption and sale
